# Dietary Polyphenols Remodel DNA Methylation Patterns of NRF2 in Chronic Disease

**DOI:** 10.3390/nu15153347

**Published:** 2023-07-27

**Authors:** Srinivasaragavan Divyajanani, Kannan Harithpriya, Kumar Ganesan, Kunka Mohanram Ramkumar

**Affiliations:** 1 Department of Biotechnology, School of Bioengineering, SRM Institute of Science and Technology, Kattankulathur 603210, Tamil Nadu, India; ds3263@gmail.com (S.D.); harithpriya2201@gmail.com (K.H.); 2 School of Chinese Medicine, LKS Faculty of Medicine, University of Hong Kong, 3 Sassoon Road, Hong Kong, China; kumarg@hku.hk

**Keywords:** hypomethylation, hypermethylation, epigenetics, nuclear factor erythroid 2-related factor 2, DNMTs, dietary polyphenols, phytochemicals

## Abstract

The nuclear factor erythroid 2-related factor 2 (NRF2) is a transcription factor crucial in regulating cellular homeostasis and apoptosis. The NRF2 gene has been implicated in various biological activities, including antioxidant, anti-inflammatory, and anticancer properties. NRF2 can be regulated genetically and epigenetically at the transcriptional, post-transcriptional, and translational levels. Although DNA methylation is one of the critical biological processes vital for gene expression, sometimes, anomalous methylation patterns result in the dysregulation of genes and consequent diseases and disorders. Several studies have reported promoter hypermethylation downregulated NRF2 expression and its downstream targets. In contrast to the unalterable nature of genetic patterns, epigenetic changes can be reversed, opening up new possibilities in developing therapies for various metabolic disorders and diseases. This review discusses the current state of the NRF2-mediated antioxidative and chemopreventive activities of several natural phytochemicals, including sulforaphane, resveratrol, curcumin, luteolin, corosolic acid, apigenin, and most other compounds that have been found to activate NRF2. This epigenetic reversal of hypermethylated NRF2 states provides new opportunities for research into dietary phytochemistry that affects the human epigenome and the possibility for cutting-edge approaches to target NRF2-mediated signaling to prevent chronic disorders.

## 1. Introduction

Nuclear factor erythroid 2-related factor 2 (NRF2), a member of the basic leucine zipper family and cap “n” collar family (CNC), plays a vital role in maintaining cellular protection against oxidative stress and inflammation. NRF2 regulates inducible and constitutive resistance to electrophiles and oxidative components [1]. Oxidative stress occurs when cells and tissues produce excessive reactive oxygen species (ROSs), and the endogenous antioxidant system cannot neutralize them [2]. In normal conditions, the mitochondrial oxidative mechanism produces ROSs and peroxides during cell respiration. Under hypoxia conditions, the cells generate excessive ROSs and reactive nitrogen species, which upon prolonged exposure, can cause injury to cellular structure and functions [3,4]. While many mechanisms quench or weaken the cellular ROSs in the cells, the need for a master controller involving a set of regulatory mechanisms with antioxidant properties is increasing. This cellular defense mechanism by NRF2 transcriptionally activates a series of antioxidant genes such as NADPH quinone oxidoreductase (NQO1), heme oxygenase (HO-1), catalases (CAT), and glutathione peroxidase (GPx). Furthermore, NRF2 regulates the inflammation genes such as transforming growth factor (TGF-β) and nuclear factor kappa (NF-kB) [5]. Studies have revealed that increased oxidative stress in renal disease impairs NRF2 function and leads to mitochondrial dysfunction and lipid damage [6]. In type 2 diabetes mellitus, the activation of NRF2 regulates oxidative stress by suppressing intracellular ROS formation and inhibiting pancreatic β-cell apoptosis [7]. This review discusses the impact of dietary polyphenols on the DNA methylation patterns of NRF2, along with how these changes aid in avoiding diseases and enhancing overall health. It provides a summary of the many phytochemicals that control NRF2 via demethylating DNA.

## 2. Structure and Regulation of NRF2

In normal conditions, Kelch-like ECH-associated protein 1 (Keap1) actin cytoskeleton-associated adapter protein of Cullin3 (Cul3-) binds to NRF2, leading to its proteasomal degradation via ubiquitination [6,8]. In NRF2, six domains show high conservation of homology (Neh1–Neh6), where Neh2 regulates the cytoplasmic localization of NRF2 and the Neh4 and Neh5 domains facilitate the recruitment of canonical protein and transcription factors responsible for expression [9]. By associating with Neh2 and Neh6 (domains of NRF2), Keap1 and β-transducin repeats containing proteins regulate NRF2 intracellularly by recruiting E3 ubiquitin ligases. The conformational modifications caused by ectopic and endogenous electrophiles in Keap1 prevent NRF2 from being degraded by Keap1. Further, the presence of 20 cysteine residues in the Keap1 protein has been shown to facilitate the redox-based stimulation of NRF2 [10], in which the cys288 and cys273 residues play a critical role in Keap1-mediated NRF2 inhibition. The mutations in these residues activate NRF2 by inhibiting the Cul3/E3/Keap1-mediated degradation.

**Figure 1:**
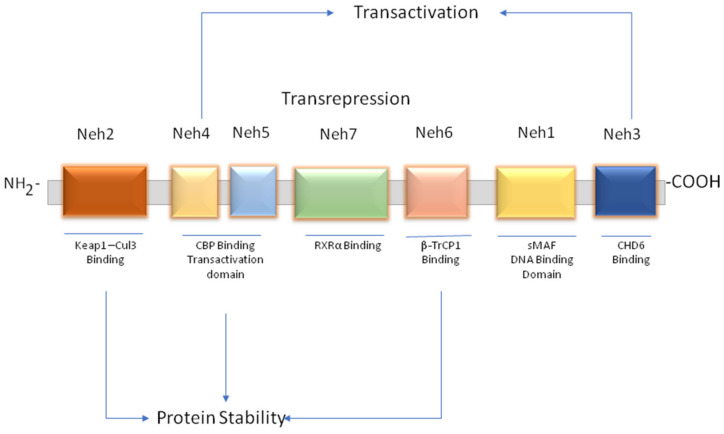
Genetic and epigenetic regulation of NRF2. Genetic regulation of NRF2 is achieved by the interaction of various transcriptional, post-transcriptional, and translation regulations, including inhibiting Keap1-mediated ubiquitination, NRF2 promoter binding by counteract molecules such as NF-kB, AhR receptor binding, and by sMafs, wherein epigenetic regulation of NRF2 involves the action of DNMTs, HDACs, and HATS, potent epigenetic markers.

Translocation of stabilized NRF2 interacts with small Maf (sMaf) and other proteins in the nucleus. It transcribes a battery of ARE-driven antioxidant genes such as glutathione peroxidase (GPx), NAD(P)H quinone oxidoreductase 1 (NQO1), superoxide dismutase (SOD), heme oxygenase-1 (HO-1), and catalases (CATs) responsible for cellular homeostasis. As an essential redox homeostatic regulator, NRF2 controls the expression of these enzymes involved in NADPH regeneration, ROS detoxification, and heme metabolism (Figure 1) [11].

### 2.1. Genetic Regulation of NRF2

NRF2 can be regulated at the genetic level by controlling the cellular process at the transcriptional and post-transcriptional levels. Various mechanisms regulating NRF2 include mRNA processing, transcriptional regulation, translation, protein stability, and sub-cellular localization.

#### 2.1.1. Transcriptional Level Regulation of NRF2

Transcriptional activation of NRF2 is regulated by factors such as xenobiotics, hypermethylation, and single-nucleotide polymorphisms (SNPs). One such genetic activation of NRF2 is through the aryl hydrocarbon receptor (AhR), which binds to the NFE2L2 promoter xenobiotics response element as a heterodimer with nuclear translocator of AhR, thereby activating its transcriptional regulation [12]. An AhR-deficient mouse hepatoma cell line showed a loss of the mRNA expression of NRF2, thereby implying that the ARE-NRF2 element is located downstream of the AhR-ARE pathway [12]. Similarly, the NRF2 promoter has the binding site of another transcription factor, NF-kB, which induces transactivation through the siRNA knockdown of p50 and p65, NF-kB subunits in acute myeloid leukemia [13]. The knockout of p65 helps promote the NRF2-CREB-binding protein (CBP), thereby decreasing the expression of NF-kB target genes such as iNOS [14]. The tumor suppressor BRCA1, a potent NRF2 binding protein, restores the stability of and activates NRF2 by inhibiting the Keap1-mediated NRF2 ubiquitination. Reports have suggested that NF-kB inhibition by NRF2 activators regulates the expression of NRF2 via PI3K/AKT signaling [15,16]. Along with this, protein kinase C (PKC) also phosphorylates the Ser40 present in the Neh2 domain of NRF2, disturbs the NRF2-Keap1 association, and enhances NRF2 expression [17,18].

MafF, MafG, and MafK are members of the bZIP transcription factor family, which bind to DNA and help regulate gene function. The sMaf protein itself lacks a transactivation domain and represses the transcription in homodimer form [19]. sMaf interacts with other CNC family proteins and NRF2, forms heterodimers, and activates downstream targets such as NQO1, HO-1, SOD, and CAT through ARE/EpRE [20]. Additionally, it has been shown that ATF4 can transcriptionally activate NRF2 by forming heterodimers and recognizing the ARE elements in the genome [21,22].

#### 2.1.2. Post-Transcriptional Regulation of NRF2

In addition to the transcription regulation, the post-transcriptional and translational regulation of NRF2 also give a critical insight into translocation and activation. One such important regulator of NRF2 is mRNA—the binding protein called HuR, whose ubiquitous nature endorses the maturation of NRF2-mRNA and promotes nuclear translocation [22]. On the other hand, along with HuR, AUF1 stabilizes the expression of NRF2 upon binding to the AU-rich elements [23,24]. On the other hand, microRNAs, short non-coding RNAs, also regulate the expression of NRF2. A study by Carolyn et al., stated that the expression of miR-144 was inversely correlated with NRF2 expression in erythrocyte cells of sickle cell disease [25]. Furthermore, a few other miRNAs, such as miR-142-5p and miR-27a, negatively affect the NRF2 levels, leading to ineffective transactivation of ARE enzymes [26]. On the other hand, miR28 decreases the expression of NRF2 by targeting the 3′UTR in breast epithelial cells [27].

#### 2.1.3. Translational Level of the Regulation of NRF2

Translation regulations of NRF2 are mediated by Keap1-Cul3 E3 proteasomal ubiquitination. Reports on the knockdown state that a decrease in the level of Keap1 protein expression results in the accumulation of NRF2 [28]. Few cytoplasmic proteins interact with NRF2-Keap1 to increase the stabilization of the protein p62, known as sequestosome protein-1, whose STGE motif is similar to the NRF2 ETGE motif, which competes with the NRF2 to bind with Keap1 [29]. The overexpression of the p62 protein in the cytoplasm increases, leading to the breakdown of Keap1, consequently activating and stabilizing the nuclear translocation of NRF2 [30,31]. Similarly, p21, a cyclin-dependent kinase inhibitor modulated by p53, interacts with Keap1 by competing with NRF2 for the DLG motif, stabilizing the transactivation and promoting NRF2-mediated antioxidant response [15].

### 2.2. Epigenetic Regulation of NRF2

In addition to genetic alterations in NFE2L2/Keap1, epigenetic changes have recently expanded the scope of NRF2 signaling. Histone modifications, DNA methylation, and microRNAs are believed to be the epigenetic mechanisms responsible for regulating NFE2L2 and Keap1. In contrast to genetic changes affecting the DNA sequence, epigenetic alterations are reversible, which makes them an attractive avenue for disease management [32].

#### DNA Methylation and NRF2

In addition to histone modification, DNA methylation plays a vital role in regulating gene expression. DNA methylation involves the transfer of the methyl group from universal donor 5-methylcytosine to the fifth position of cytosine [33]. The pattern of DNA methylation in the genome changes during development due to the result of both de novo and demethylation processes. DNA methylation is mainly controlled by the DNA methyltransferase enzymes (DNMTs), namely DNMT1, DNMT3a, and DNMT3b. The pattern of DNA methylation is determined by de novo DNMT3a and DNMT3b during embryogenesis and development and then maintained by the de novo DNMT1 during DNA replication in mammalian cells [34]. In human genes, almost 60% is clustered with CpG islands in their promoter region, whose expression can be altered epigenetically by DNMTs. In addition, DNMTs can act along with histone deacetylases (HDACs) and histone methyltransferase (HMTs) to regulate the gene expression of potent transcription factors in preventing diseases [35]. Hypermethylation of these CpG islands in the promoter region tends to lower the expression of NRF2, which is associated with disease progression [35]. Several studies have reported that the altered DNA methylation pattern in the NRF2 promoter corresponds to oxidative-stress-induced disease pathogenesis. For instance, studies conducted by Zhao et al., stated that the expression of NRF2 in the Alzheimer’s disease model was reduced due to the action of the DNA methyltransferase enzyme [36].

**Figure 2:**
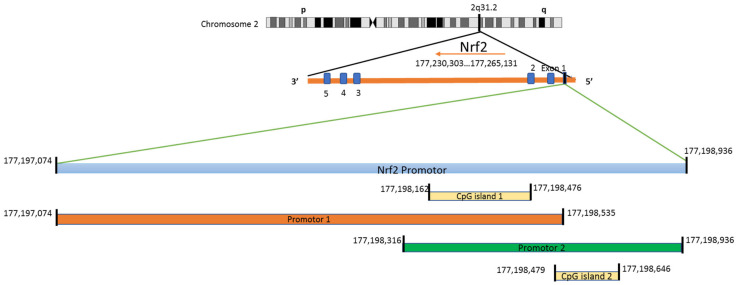
Linear graphical map of NRF2 promoter region. The human NRF2 gene is located in chromosome 2 at cytogenic band 2q31.2 spanning 178,095,031 bp to 178,129,859 bp. NRF2 has 2 promoter regions where promoter 1 has a length of 1461 bp and promoter 2 a length of 620 bp. The only region to study DNA methylation CpG island encompassing the transcriptions’ start site serves as the potential target in epigenetics. CpG island 1 of the NRF2 promoter spans around 314 bp, and CpG island 2 spans around 167 bp, which are potent targets in unraveling DNA methylation, an epigenetic target.

On the other hand, DNMT1 and DNMT3a potentially methylate the first 15 CpG sites in the Nfe2l2 promoter region, reducing the expression of NRF2 in mouse skin epidermal cell JB6P [37]. Antioxidant compounds often modulate DNA methylation at CpG sites in the promoter region to regulate NRF2 and its downstream target expression (Figure 2) [38]. Epigenetic regulation during disease pathogenesis can be reversed or prevented by NRF2 modulators, unlike genetic mutations [39]. As a result, pharmacologically targeting epigenetic events has emerged as a promising method for treating or preventing a wide range of diseases. One possible target is using dietary phytochemicals acting at various transcription levels, post-transcription, and post-translation, which could lead to novel disease prevention approaches [40,41].

## 3. Role of NRF2 in Diseases

A wide range of experimental and observational studies have established the incontestable role of NRF2 in the prevention and treatment of various diseases. Growing evidence indicates that decreased NRF2 activity contributes to oxidative stress, favoring the pathophysiology of multiple diseases, including cardiovascular disorders (CVDs) in obesity, diabetes mellitus, and atherosclerosis [42]. The systemic administration of specific NRF2 inducers benefits cardiovascular diseases. Cardiovascular health depends on the condition of vascular tone [43]. The endothelium, the main regulator of vascular homeostasis, has proven to be dysregulated in diseases such as CVDs and atherosclerosis [44]. Endothelium dysfunction is characterized by several factors, such as an imbalanced production of factors responsible for vasodilation, vasoconstriction, and elevated ROSs [45]. In this line, Amin et al., identified a lessened expression of NRF2 on human endothelial cells exposed to Thapsigargin, which stimulates endoplasmic reticulum (ER) stress. Rosolic acid, a potential NRF2 activator, was demonstrated to alleviate ROSs, which triggers the increased accumulation of ROSs in human endothelial cells under ER stress [46]. Further, a specific role of NRF2 in regulating the ER stress response was established in this study using CRISPR knockout endothelial cells. Moreover, the activation of NRF2 through Rosolic acid was established to alleviate endothelial dysfunction under the pancreatic microenvironment using a co-culture setup [47].

It has been described that NRF2 also plays a vital role in renal protection against oxidative stress in renal diseases [48]. The activation of NRF2 has been shown to inhibit the production of pro-inflammatory cytokines and promote the generation of anti-inflammatory molecules. This modulation of the immune response can help attenuate the autoimmune-driven inflammation associated with these disorders. This activation of NRF2 restores insulin expression and glycolysis, thereby inhibiting gluconeogenesis [49]. An immunomodulatory role for NRF2 has recently gained appreciation as it has been shown to protect cells and hosts alike in various immune and inflammatory disorders [50]. It has also been reported that several activators of NRF2 are involved in improving the prognosis of liver diseases by inhibiting the expression of pro-inflammatory cytokines, simultaneously inhibiting the ROS production and M1 phenotype in inflammatory disorders [51]. Macrophages are one of the notable players of inflammation in wound healing. Victor et al., recently discussed the role of NRF2 in diabetic wounds, where the incontestable role of NRF2 in promoting impaired wound healing was reported for the activation of NRF2 signaling [52]. Dysregulation of NRF2 in macrophages has been reported to cause delayed healing in diabetic wounds. We provided evidence of the dysregulation of NRF2 in macrophages under a diabetic environment in vitro [53]. Further, we evidenced the restoration of NRF2 signaling and the impairment of macrophage function upon treatment with pterostilbene, a stilbenoid pharmacological compound. In addition to this, we identified the effect of pterostilbene on the polarization of M2 macrophages through NRF2 signaling under a diabetic stimulus in vitro [54].

The liver, the central storage organ for vitamins and elements such as iron and copper, plays a vital role in many physiological activities. Besides its role in bile breakdown, the liver is involved in synthesizing and metabolizing proteins, fats, etc. [55]. The association of NRF2 in liver homeostasis and the accumulation of ROSs in liver injury has been well studied. Numerous molecular mechanisms such as oxidative stress are associated with liver injury, which disturbs the balance between ROSs and antioxidant enzymes such as glutathione [56]. For instance, Li et al., demonstrated CCl_4_-induced liver damage in rats, which was found to reverse upon administration of ginsenoside Rg1 [57]. Another study showed the progression of metabolic dysfunction associated with fatty liver diseases (MAFLD) on the knockout of NRF2, which decreased glutathione levels. On the other hand, pharmacological compounds such as scutellarin, apigenin, osteocalcin, and berberine were found to improve MAFLD by activating NRF2 signaling [56].

NRF2, a double-edged sword, is foreseen to have a role as a tumor progressor in many cancers, but a handful of evidence suggests NRF2’s role against tumorigenesis. NRF2, activated by BRCA1, increased phase-II enzymes and abrogated tumor progression. For instance, NRF2-deficient mice were found to have aggravated oxidative stress with decreased levels of antioxidant enzymes such as GST and NQO1 [58]. A study by Ramos-Gomez et al., observed a larger number of tumor development in NRF2-deficient mice compared to the wild-type, emphasizing the importance of NRF2 in antitumor activity [59].

NRF2 deficiency increases renal injury, loss of kidney function, oxidative and reticulum endoplasmic stress, and cell death. Several small-molecule NRF2 activators are currently in clinical trials in different disease settings. A few reports have highlighted that NRF2 activators improve cognitive function, metabolic health, and longevity [60].

Overall, NRF2 plays a dual role in diseases. While its activation can be a benefit by promoting antioxidant defenses and reducing inflammation, abnormal or dysregulated NRF2 activity can also contribute to disease progression in specific contexts. Therefore, pharmacologically activating NRF2 for combating oxidative stress and inflammation for chemoprevention and intervention in other chronic diseases, including neurodegeneration, diabetes, and cardiovascular disease, remains an important aspect [61]. The regulation of the NRF2 pathway is of particular interest to better understand how the context and mechanisms of disease affect NRF2 function. Recently, it has been found that epigenetics is one important potential mechanism for regulating the NRF2 pathway, and a few phytochemicals have been found as NRF2 epigenetic modifiers.

## 4. Role of Dietary Polyphenols in Epigenetic Modulation

A wide range of plant-based active compounds have been reported to possess various disease-prevention and medicinal properties. Numerous long-term studies have shown associations between consuming phytochemicals and lowering the risk of cardiovascular, neurodegenerative, inflammatory, microbial infections, and metabolic diseases. For example, according to a survey in a 2011 study, individuals in the top 20% of the study group who regularly consumed the most vegetables seemed to have a 16% decline in all-cause mortality than many other individuals in their age groups during the study period [62]. Studies have also found that people who consume more vegetables have a lower risk of developing cancer [63]. These health benefits might be attributed to the enormous chemical compounds in plant-based foods. Regular intake of bioactive compounds in plant-based foods, such as polyphenols, isothiocyanates, sulfur-containing compounds, and terpenoids (such as carotenoids), has been related to disease prevention [64].

A category of compounds that provide an array of health benefits is dietary polyphenols. These are bioactive compounds found in plant-based foods containing a wide range of chemical structures derived from flavonoids, lignans, stilbenes, and phenolic acids. Dietary polyphenols are the most-abundant antioxidants in the human diet and perform a wide range of biologically essential tasks, including preventing oxidative stress and degenerative illnesses. According to experimental evidence, most of these biological activities can be linked to their innate antioxidant capacities. They are abundant in edible herbs and spices such as star anise, celery seed, rosemary, cinnamon, and cloves. They are also widely present in fruits, grains, and vegetables such as berries, peach, flax seeds, almonds, red onion, and spinach [65,66]. Latterly, polyphenols have gained researchers’ interest owing to their antioxidant properties, which protect against reactive oxygen species (ROSs) and help to prevent oxidative stress-related pathological conditions or diseases (ROS) [67].

ROSs can cause oxidative damage to biomolecules, including lipids, proteins, and DNA. High levels of ROSs have been linked to the development of aging and several illnesses, including cancer, respiratory, cardiovascular, neurological, and digestive diseases [68]. Polyphenols can neutralize free radicals by donating an electron or hydrogen atom. For the antioxidant properties, there are highly conjugated systems and specific hydroxylation patterns, such as the 3-hydroxy group in flavonols [69]. In addition to their anti-inflammatory and antibiotic properties, polyphenols can also induce the nuclear transcription factor NRF2, which protects against oxidative stress and inflammation [70]. Polyphenols may help moderate inflammation levels in various ways, such as targeting signaling pathways and the reduction of reactive oxygen and reactive nitrogen species.

**Figure 3:**
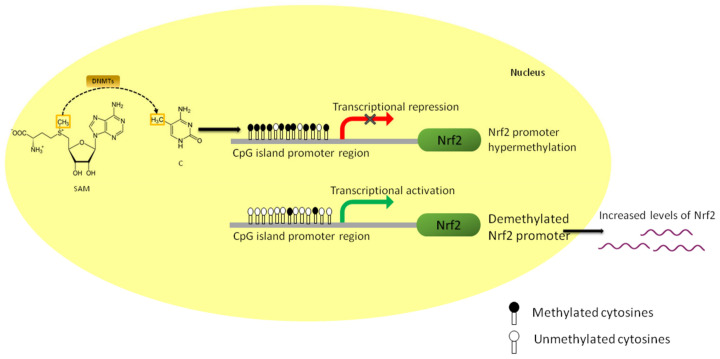
Schematic overview of the role of DNA methylation in NRF2 transcription. DNA methyltransferase (DNMT) enzymes add methyl groups of the CpG islands in the promoter region of NRF2. The hypermethylation of the CpG islands inhibits the binding of the transcription factor(s) to the DNA, which transcriptionally represses the NRF2 gene. By demethylating the promoter, transcription factors bind to the NRF2 sequence and initiate transcription, thereby increasing the NRF2 expression levels.

**Figure 4:**
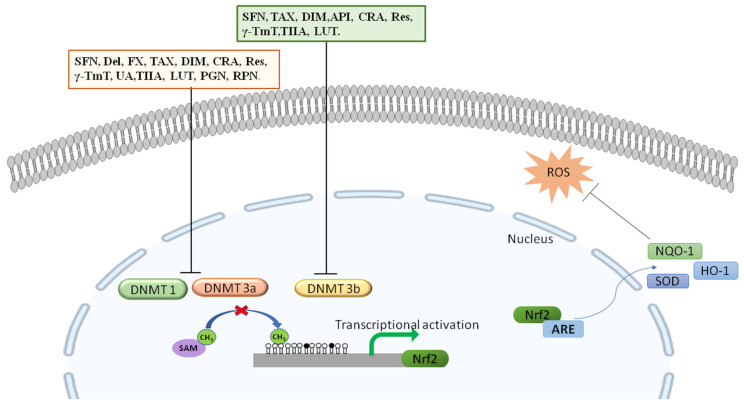
The activity of dietary polyphenols on NRF2 and its downstream targets. The expression of DNA methyltransferase variants DNMT1 and DNMT3a is inhibited by SFN, Del, FX, TAX, DIM, CRA, Res, γ-TmT, UA, TIIA, LUT, PGN, and RPN, whereas the variant DNMT3b is inhibited by SFN, TAX, DIM, API, CRA, Res, γ-TmT, TIIA, and LUT. Inhibition of DNMTs prevents the methylation of NRF2, thereby increasing its expression, and under conditions of excessive ROSs, NRF2 translocates into the nucleus to initiate the transcription of its downstream targets such as HO-1, NQO-1, and SOD, which combat oxidative stress. White circle indicates the non-methylated cytosine. Black circles indicates methylated cytosine.

Even though DNA methylation is crucial for healthy physiological functions, different and aberrant DNA methylation patterns at specific locations may silence the gene by blocking transcription, thereby altering the function of that specific gene [71]. In cancer cells, the DNA methylation ratio of the NRF2 gene promoter region is inversely proportional to the protein and mRNA expression of NRF2 and its downstream genes [72]. Therefore, as discussed earlier, DNA methylation contributes to the suppression of NRF2 signaling, which can be restored by the action of phytochemicals in demethylation (Figure 3). Few phytochemicals have been reported to modulate NRF2 signaling by inhibiting DNA methyltransferases (DNMTs) [73]. This epigenetic reversal of hypermethylated NRF2 states provides new opportunities for research into dietary phytochemicals that affect the human epigenome and the possibility for cutting-edge approaches to target NRF2 signaling to prevent chronic disorders. Several compounds are known to activate NRF2 epigenetically; their mechanisms and effects on NRF2 are briefly discussed below (Table 1) (Figure 4).

## 5. DNA Hypomethylation of NRF2 by Phytochemicals

### 5.1. Sulforaphane

Sulforaphane (1-isothiocyanate-(4R) (methylsulfonyl)butane) (SFN) is a naturally occurring isothiocyanate formed by the enzymatic action of myrosinase on glucopharanin, a 4-methyl-sulfinyl butyl glucosinolate found in Brassica cruciferous vegetables such as broccoli, Brussels sprouts, and cabbage. SFN stimulates the production of several cytoprotective proteins, including antioxidant enzymes, by regulating the NRF2-antioxidant response element pathway [74]. According to Kubo et al., 2017, SFN attenuates the loss of Prdx6 expression caused by impaired ARE/NRF2 regulation to protect against diseases associated with aging [93].

In a study aimed at knowing the NRF2 restoration potential in TRAMP C1 prostate cells, SFN treatment was found to demethylate DNA in its 5 CpGs in the promoter region of the NRF2 gene and increase the expression of the downstream target gene NQO-1 in both mRNA and protein. SFN also reduced the levels of DNMT1 and DNMT3a proteins, increasing NRF2 levels [94]. Another study designed to determine sulforaphane’s neuroprotective effects in the disease model of Alzheimer’s disease revealed that sulforaphane upregulated NRF2 expression and promoted NRF2 nuclear translocation via decreasing DNA methylation levels of the NRF2 promoter in mouse neuroblastoma Appswe cells. Sulforaphane and 5-Aza-dC reduced the protein levels of DNMT1, DNMT3a, and DNMT3b. It also increased the mRNA and protein levels of NRF2, NQO1, and HO-1. Sulforaphane inhibited oxidative stress via NRF2 upregulation in the Alzheimer’s disease cell line [36].

SFN was also reported to inhibit protein levels of DNMT1, reduce NRF2 promoter DNA methylation, and increase mRNA and protein levels of NRF2, which resulted in the prevention of the neoplastic transformation of TSA-induced Caco-2 cells, which suggests that NRF2 might have a chemopreventive effect against human colon cancer.

### 5.2. Reserpine

Reserpine is an indole alkaloid polyphenol extracted from Rauwolfia serpentine roots. It has been used for centuries as traditional Chinese medicine (TCM) to treat hypertension, mental conditions, blood pressure, snake bites, inflammation, and pruritus, among other diseases. In a study by Al-Qirim et al., it was found that Rauvolfia verticillate extract protects mouse cardiomyocytes from oxidative free radical damage [95]. In another study, reserpine inhibited the neoplastic transformation of JB6 P+ cells through epigenetically activating NRF2 and its downstream targets. Treating JB6 P+ cells with reserpine decreased the mRNA and protein levels of epigenetic enzymes such as DNMT1, DNMT3a, and DNMT3b and increased the levels of NRF2 downstream targets HO-1, NQO1. Reserpine reduces the proportion of methylated CpG sites in the NRF2 promoter and can alter DNA demethylation and epigenetically boost NRF2 expression, thereby reducing redox stress [96].

### 5.3. Fucoxanthin

Fucoxanthin is a carotenoid in microalgae and macroalgae, such as brown seaweeds. According to recent research, fucoxanthin contains several physiological functions, including antiobesity, anticancer, antidiabetes, antioxidant, anti-inflammatory, hepatoprotective actions, and cardiovascular and cerebrovascular protective benefits [97]. As a result, fucoxanthin can be utilized medicinally and nutritionally to prevent and arrest the progression of chronic disorders [98]. In JB6 P+ cells, FX reduced the methylation of the NRF2 promoter region. FX activates the NRF2 signaling pathway, causes epigenetic DNA demethylation of CpG sites in NRF2, and increases the protein expression levels of NRF2, NQO1, HO-1, and SOD in a concentration-dependent manner, thereby reducing oxidative stress [79].

### 5.4. Luteolin

Luteolin is a flavonoid in various plants, such as broccoli, pepper, thyme, and celery. Multiple studies have shown that luteolin has neuroprotective properties both in vitro and in vivo. Luteolin has many biological activities, including antioxidant, anti-inflammatory, antimicrobial, and anticancer properties [99]. Luteolin’s capacity to inhibit angiogenesis, induce apoptosis, prevent carcinogenesis in animal models, reduce tumor growth in vivo, and sensitize tumor cells to the cytotoxic effects of some anticancer drugs suggests that it has cancer chemopreventive and chemotherapeutic potential. The biological activities of luteolin also include combating high ROS levels [99]. In human colon cancer cells, HT-29, luteolin promotes DNA demethylation of the NRF2 promoter, inhibits the expression of DNA methyltransferases, and increases the levels and activity of ten-eleven translocation (TET). Luteolin reduces the methylation of the NRF2 promoter region, increasing NRF2 mRNA expression. Furthermore, luteolin increases TET1 binding to the NRF2 promoter [100]. In HCT116 cells, luteolin reduces the amount of methylation in the NRF2 promoter area, following the enhanced mRNA production of NRF2, increasing the mRNA and protein expression of NRF2, HO-1, and NQO1, which shows that luteolin subsequently induces the downstream antioxidant pathway. It also reduces the expression of DNMT1, DNMT3a, and DNMT3b [79].

### 5.5. Pelargonidin

Pelargonidin is a natural phenolic pigment found in berries, strawberries, blueberries, red radishes, and other natural foods and has been shown to possess anticancer properties [101]. In a study aimed to investigate how pelargonidin affects the cellular transformation in JB6 cells, pelargonidin reduced DNA methylation in the NRF2 promoter region and increased the protein and mRNA expression of the protein levels of the NRF2 target genes HO-1 and NQO1 [80].

### 5.6. Tanshinone IIA

Tanshinone IIA is one of the many lipophilic active ingredients in the traditional Chinese herb *Salviae miltiorrhiza*, used to relieve pain, improve blood circulation, etc. Recently, Tanshinone IIA has been reported to have anticancer, anti-inflammatory, and antioxidative properties [102]. An earlier study found that NRF2 is involved in the cytoprotective effects of Tanshinone IIA by lowering intracellular redox status and defending against oxidative stress via the ERK and PKB signaling pathways in human aortic smooth muscle cells [103]. In a study that further tried to elucidate the mechanism by which TIIA reduced oxidative stress in JB6 P+ cells, an epidermal cell line, it was discovered that TIIA regulates NRF2 activation epigenetically by decreasing promoter DNA methylation. TIIA-induced NRF2 targets genes in mouse epidermal JB6 cells to inhibit TPA-stimulated neoplastic transformation. TIIA increases the mRNA and protein levels of NRF2 target enzymes HO-1 and NQO1. TIIA treatment considerably inhibits both the mRNA and protein levels of DNMTs (DNMT1, DNMT3a, and DNMT3b). Considering that some HDACs may be involved in the DNMT complex, which mediates DNA methylation [104], TIIA’s capacity to inhibit HDACs could also contribute to activating NRF2. The inhibitory effect of TIIA on these epigenetic modification enzymes induces NRF2 activity in JB6 P+ cells via DNA demethylation of the NRF2 gene promoter, indicating a potential role in skin cancer chemoprevention [82]. The effect of Tanshinone IIA, inducing NRF2 epigenetically by increasing the expression of TET2, was found to be beneficial in preventing RFP-induced liver injury in hepatocytes [83].

### 5.7. Delphinidin

Delphinidin is an anthocyanidin found in pigmented vegetables, fruits, and berries, exhibiting intriguing antioxidant and anti-inflammatory characteristics [105]. In JB6 P+ cells, Delphinidin decreases the CpG DNA methylation ratio in the NRF2 promoter, increases the mRNA and protein levels of HO-1 and NQO1 and the reactive oxygen species (ROS) scavenger SOD1, and downregulates the protein expression of DNMTs (DNMT1 and DNMT3a) in a dose-dependent manner [76].

### 5.8. Ursolic Acid

Ursolic acid (UA), a natural pentacyclic triterpenoid carboxylic acid, is a crucial constituent of several traditional medicinal herbs and is widely known to have a variety of biological activities, including antioxidative, anti-inflammatory, and anticancer properties [106]. UA can reduce the toxic effects of reactive oxygen species (ROSs) and enhance the activity of antioxidant enzymes. In human skin cells, ursolic acid effectively inhibits UVA-modulated signal transduction pathways such as ROS production, lipid peroxidation, MMP-2 expression, and DNA damage in human keratinocyte HaCaT cells [107]. Recent research has shown that UA protects the brain from cerebral ischemia in mice via activating the NRF2 pathway [108]. UA was also demonstrated to protect against liver fibrosis by activating the NRF2 pathway [109]. Furthermore, in a study aimed to analyze the effect of ursolic acid on TPA-induced mouse epidermal cells, UA reduced NRF2 promoter DNA methylation and negatively regulated epigenetic modification enzymes such as DNMT and HDACs. The NRF2 target enzymes HO-1 and NQO1 have their mRNA and protein levels increased by UA. UA inhibits the TPA-induced transformation of JB6 P+ cells by boosting anti-inflammatory and antioxidant enzymes, which is mediated by increased NRF2 expression [84].

### 5.9. γ-TmT

The γ-tocopherol-rich mixture of tocopherols (γ-TmT) is a byproduct of the bio-refinery industry in soybean oil production. It consists of 57% γ-tocopherol, 24% δ-tocopherol, 13% α-tocopherol, and 1.5% β-tocopherol. Earlier studies have suggested that γ-TmT has anti-inflammatory and anticancer properties against different cancers [110,111,112]. Moreover, in a recent study, γ-TmT treatment reversed the methylation of the first five CpG in the NRF2 promoter, with consequent lower DNMT protein expression, and induced the mRNA and protein expressions of NRF2 and NQO1 in prostate tissues of C57BL/TGN TRAMP mice, all of which contribute to increased NRF2 expression, which may play a role in the prevention of prostate tumorigenesis through epigenetic mechanisms [85].

### 5.10. Resveratrol

Resveratrol is a naturally occurring polyphenolic phytoalexin in various foods such as grapes, wine, peanuts, and soy [113]. It has many biological properties, including antioxidant, detoxification, anti-inflammatory, and anticancer properties. It has high anticancer activity, affecting transcription factors such as p53/p21 and IkB kinase/NF-kB. Resveratrol inhibited the DNA-binding activity of NF-kappaB in MCF7 cells and chemically induced rat mammary tumors [114]. Resveratrol has been demonstrated in animal models of breast cancer to prevent drug-induced mammary cancer progression and to change breast development and morphology [115]. Several studies that aim to reverse aging have suggested that resveratrol acts as an epigenetic modifier [116].

In a research study on HepG2 cells subjected to high glucose and in high-fat NAFLD models, it was found that the methylation status of the NFE2L2 gene increased, whereas that of Keap1 decreased, resulting in reduced NRF2 expression and activity. The administration of resveratrol led to the increased mRNA and protein expression of NRF2, HO1, NQO1, and SOD. In HFD-fed mice, resveratrol could reverse the DNA methylation pattern in the NRF2 promoter. Resveratrol suppressed the DNMT1, DNMT3a, and DNMT3b levels in liver tissue and HepG2 cells. Resveratrol reduces oxidative stress and lipid accumulation by demethylating the NRF2 signaling pathway [86]. An earlier study using an estrogen-induced mammary cancer rat model found that resveratrol affected the DNA methylation status of the NFE2L2 promoter. Resveratrol treatment significantly increased the mRNA and protein expression of NRF2 and NRF2-mediated cancer-protective phase-II enzymes such as NQO1, SOD3, and OGG1. Resveratrol inhibits cell proliferation while inhibiting oxidative DNA damage and the carcinogenic process in the mammary gland [87].

### 5.11. Curcumin

Curcumin is the biphenolic active compound present in turmeric. Curcumin is one of the most-potent polyphenols and has been shown to have multiple molecular targets attributed to its immense therapeutic potential. Curcumin has been found to help with various inflammatory conditions, metabolic syndromes, pain relief, degenerative eye conditions, and renal diseases due to its antioxidant and anti-inflammatory properties [117]. Another study reported that prolonged exposure to curcumin induces phase-II antioxidant enzymes by activating NRF2 signaling and restores phase-II antioxidant enzymes such as GST, GR, and NQO1 in the liver of lymphoma-bearing mice [118]. Recently, in a study aimed at investigating the potential of curcumin to prevent the progress of prostate cancer by epigenetically activating NRF2, it was reported that curcumin demethylated NRF2, and it was correlated with the restoration of both the mRNA and protein levels of NRF2 and its target gene, NQO-1, a key enzyme that combats antioxidative stress. The study concluded that CUR may exert its prostate cancer chemopreventive effect by epigenetically modifying the NRF2 gene, activating the NRF2-mediated antioxidative stress cellular defense pathway. Two synthetic curcumin derivatives, E10 and F10, have been demonstrated to activate NRF2 by reducing the rate of methylation of the CpG regions in the NRF2 promoter and increasing protein and mRNA expression levels of NRF2 downstream targets HO-1, NQO1, and UGT1A1 [119].

### 5.12. Z-Ligustilide

Z-Ligustilide is a benzoquinone, which is a naturally found active compound in many herbs such as ginseng, Ligusticum chuanxiong Hort, *Ligusticum sinense* Oliv., and Ligusticum jeholense. It possesses many therapeutic properties such as anticancer, neuroprotective, antihepatotoxicity, and anti-inflammatory effects. It has also been known to relieve menstrual pain, help with postpartum blood deficiency and headaches, and even treat coronary heart diseases [120]. *Z*-Ligustilide in an ethanol extract of *LC* rhizome inhibited oxidative stress by acting upon the NRF2 and NF-κB pathways [121]. A study that analyzed the effect of Z-Ligustilide in the murine prostate cancer cell line reported that it increases NRF2 mRNA and protein expression in TRAMP C1 cells by reducing the expression of DNMTs and demethylating the DNA in the NRF2 gene promoter. The restoration induces the NRF2 downstream target genes, such as phase-II detoxifying enzymes such as HO-1, NQO1, and UGT1A1, which help alleviate oxidative stress [89].

### 5.13. Corosolic Acid

Corosolic acid is a pentacyclic triterpene compound found in various sources such as *Eriobotrya japonica*, *Lagerstroemia speciosa* L., *Orthosiphon stamineus*, *Actinidia chinensis*, and *Weigela subsessilis*. This compound has been proven to be effective against a range of ailments and metabolic disorders such as diabetes, obesity, and atherosclerosis [122,123,124]. In recent times, it has been gaining research due to its anti-carcinogenic properties against several types of cancers such as lung adenocarcinoma [125], cervical cancer [126], and hepatocellular carcinoma [127]. One study that was designed to study the effects of corosolic acid on prostate cancer cell lines found that corosolic acid specifically induced NRF2 transcriptionally by decreasing CpG DNA methylation in the NRF2 promoter region and the protein levels of the epigenetic enzymes DNMTs in TRAMP-C1 cells, which led to the increase of the mRNA and protein levels of antioxidant enzymes such as HO-1 and NQO1. Thus, corosolic acid potentially demethylates NRF2, thereby activating it [90].

### 5.14. Apigenin

Apigenin, 4′,5,7-trihydroxy flavone, is among the most-abundant phenols in plants and has been subjected to considerable scrutiny due to its therapeutic potential. Apigenin is found in a glycosylated form in various foods such as parsley, celery, onions, chamomile, thyme, oregano, and basil and plant-based drinks such as tea, beer, and wine [128]. Apigenin has been demonstrated to have exceptional anti-inflammatory, antioxidant, and anti-carcinogenic properties and neuroprotective functions [129,130]. A recent study found that apigenin decreased the DNA methylation status of the NRF2 promoter and inhibited the expression of DNMTS and HDACS, which restored the NRF2 expression and increased NQO1 expression levels in the preneoplastic JB6 P+ cell line, demonstrating its activity against skin cancer [91].

### 5.15. 3,3′-Diindolylmethane

3,3′-Diindolylmethane (DIM) is an indole alkaloid derivative found in many cruciferous vegetables. It has been subjected to numerous studies, implying its potential as a chemoprotective compound against many cancers [131,132]. DIM elevated NRF2 mRNA in TRAMP-C1 prostate cancer cells by inhibiting the mRNA and protein expression of DNMT1, DNMT3a, and DNMT3b. It reduced the proportion of DNA methylation in the first five CpGs in the NRF2 promoter and restored NRF2 levels, thereby increasing NRF2-target genes such as NQO1. DIM supplementation in the diet reduced the occurrence of palpable tumors and lymph node metastasis and suppressed prostate cancer tumor progression in TRAMP mice [92].

### 5.16. Taxifolin

Taxifolin, also known as dihydroquercetin, is an active flavonoid compound in various foods, including olive oil, grapes, citrus fruits, onions, and herbs. Taxifolin demonstrated a wide range of pharmacological and biochemical effects, including hepatoprotective, anti-diabetic, cardioprotective, antitumor, neuroprotective, and anti-inflammatory effects, as well as being beneficial in Alzheimer’s disease prevention [133]. A new study suggests that taxifolin prevents skin carcinogenesis through a novel molecular mechanism, where NRF2 is activated by inhibiting the protein expression of epigenetic modification enzymes such as DNMT1, DNMT3a, and DNMT3b, which in turn reduces DNA methylation in CpGs in the NRF2 gene promoter region in JB6 P+ Cells [37].

## 6. Conclusions

Over the last few years, many studies have revealed that epigenetic mechanisms are linked to controlling and regulating most biological processes in the body. Even though these epigenetic regulations are required for growth and development, they may also lead to diseases and metabolic disorders [134]. However, some epigenetic marks are reversible, which has prompted many researchers to focus on epigenetic therapy [135]. Hypermethylation of the promoter region could significantly affect the expression of a gene or even silence it. NRF2, a master regulator of antioxidant enzymes, helps produce phase-II detoxification enzymes to maintain cellular redox homeostasis. The disruption of this redox homeostasis is reported in many health conditions such as cancers, cardiovascular diseases, diabetes, aging-related problems such as Alzheimer’s disease and other neurodegenerative disorders, cognitive impairment, and frailty [136]. Hence, it becomes crucial to discover a mechanism to restore the oxidative imbalance. Recently, studies have revealed that plant-based dietary components can affect gene expression through epigenetic modifications. Compounds such as sulforaphane, resveratrol, curcumin, luteolin, corosolic acid, apigenin, and most other compounds discussed in this review activated NRF2 by inhibiting the epigenetic enzymes DNMTs. All these studies focused on the ability of the polyphenolic compound to reverse the hypermethylation states of CpGs in the NRF2 promoter, thereby increasing NRF2 levels. With further research, this ability to reverse epigenetic changes can be harnessed to treat various ailments where oxidative stress plays a key role.

## Figures and Tables

**Table 1:** List of phytocompounds and their demethylation activity.

Phytocompounds	Phytocompounds Sources	Targets	Demethylation Activity of the Compounds	References
Sulforaphane	Cruciferous vegetables	DNMT1, DNMT3a	Sulforaphane demethylates the first 5 CpGs in the NRF2 promoter region, decreases DNMT1 and DNMT3a proteins, and increases downstream target gene NQO-1 in TRAMP C1 prostate cells	[74]
		DNMT1, DNMT3aDNMT3b	Sulforaphane decreases the protein levels of DNMT1, DNMT3a, and DNMT3b, increasing the mRNA and protein levels of NRF2, NQO1, and HO-1 in the Alzheimer’s disease model	[36]
		DNMT1	Decreases protein levels of DNMT1, increases NRF2 levels, and prevents the neoplastic transformation of caco-2 cells	[75]
Delphinidin	Red fruits, some cereals, aubergines, beans, cabbages, radishes, and onions	DNMT1, DNMT3a	Delphinidin decreases CpG methylation of the NRF2 promoter region, reduces protein expression of DNMT1 and DNMT3a, and increases protein and mRNA expression of HO-1, NQO-1, and SOD-1 in JB6 P+ cells	[76]
Fucoxanthin	Brown seaweed	DNMT1, DNMT3a	Fucoxanthin downregulates DNMT1 and DNMT3a protein expression, increases HO-1, NQO-1, and SOD-1 protein and mRNA expression, and decreases CpG methylation of the NRF2 promoter region in JB6 P+ cells	[77]
Luteolin	Celery, parsley, broccoli, onion leaves, carrots, peppers, cabbages, apple skins, and chrysanthemum flowers	DNMT1, DNMT3aDNMT3b	DNMT1, DNMT3a, and DNMT3b expression is downregulated, NRF2 methylation reduced, and increases TET1 binding to the NRF2 promoter in HT-29 cells	[78]
		DNMT1, DNMT3aDNMT3b	DNMT1, DNMT3a, and DNMT3b protein levels are decreased by luteolin treatment, while NRF2, NQO1, and HO-1 mRNA and protein levels are increased, all of which correspond to the reduction in NRF2 promoter methylation in HCT116 cells	[79]
Pelargonidin	Berries, strawberries, blueberries, red radishes	DNMT1, DNMT3a	Pelargonidin increases protein and mRNA expression of HO-1, NQO-1, and SOD-1 in JB6 P+ cells while decreasing CpG methylation of the NRF2 promoter region and DNMT1 and DNMT3a protein expression in JB6 P+ cells	[80]
Reserpine	Rauwolfia serpentine roots	DNMT1, DNMT3a	Reserpine decreases CpG methylation of the NRF2 promoter region and decreases the expression of DNMT1 and DNMT3a protein in JB6 P+ cells while increasing the protein and mRNA expression of HO-1, NQO-1, and SOD-1	[81]
Tanshinone IIA	Salvia miltiorrhiza	DNMT1, DNMT3aDNMT3b	Tanshinone IIA treatment decreases methylated CpGs in the NRF2 promoter; DNMT1, DNMT3a, and DNMT3b mRNA and protein levels decrease in JB6 P+ cells	[82]
		TET2	Ten-eleven translocation 2 (TET2) is expressed as a result of TAN IIA, which mediates the demethylation of NRF2 and protects against RFP-induced cholestatic liver injury	[83]
Ursolic acid	Rosemary, marjoram, lavender, thyme, organum, and apple fruit peel	DNMT1, DNMT3a	UA reduces the expression of epigenetic modifying enzymes, including the DNA methyltransferases DNMT1 and DNMT3a and the histone deacetylases, reduces NRF2 promoter methylation, and increases the expression of HO-1 and NQO-1 in JB6 P+ cells	[84]
A γ-tocopherol-rich mixture of tocopherol	Nuts, seeds, and vegetable oils	DNMT1, DNMT3a DNMT3b	γ-tocopherol reduces DNMT1, DNMT3a, and DNMT3b protein levels and reverses hypermethylation of the Nfe2l2 promoter in C57BL/TGN TRAMP mice’s prostate tissues	[85]
Resveratrol	Grapes, wine, peanuts, and soy	DNMT1, DNMT3aDNMT3b	Resveratrol increases mRNA and protein expression of NRF2, HO1, NQO1, and SOD and lowers levels of DNMT1, DNMT3a, and DNMT3b in liver tissue and HepG2	[86]
			Hypomethylates first 5 CpGs in the NRF2 pathway and induces re-expression of NRF2, NQO-1, SOD3, and OGG1 in estrogen-induced mammary cancer rat model	[87]
Curcumin	Turmeric		CUR reverses the methylation status of the first 5 CpGs in the promoter region of the NRF2 gene and increases mRNA expression levels of HO-1, NQO1, and UGT1A1; CUR treatment does not affect both mRNA and protein levels of DNMT1, 3A, and 3B.	[88]
Z-Ligustilide	Ligusticum striatum, Angelica sinensis		Z-Ligustilide demethylates the first five CpGs of the NRF2 promoter, resulting in re-expression of NRF2 and increased HO-1, NQO1, and UGT1A1 mRNA expression in TRAMP C1 cells; Z-Ligustilide does not affect both mRNA and protein levels of DNMT1, 3A, and 3B.	[89]
Corosolic acid	Guava, loquat, and olive	DNMT1, DNMT3aDNMT3b	The NRF2 gene is re-expressed, and the expression of HO-1, NQO1, and UGT1A1 mRNA in TRAMP C1 cells is increased as a result of corosolic acid treatment, which reduces the protein levels of DNMT1, DNMT3a, DNMT3b and the demethylation of the first five CpGs in the NRF2 promoter	[90]
Apigenin	Parsley, chamomile, celery, vine spinach, artichokes, and oregano	DNMT1, DNMT3b	Apigenin demethylates 15 CpGs in NRF2 dose-dependently and enhances NRF2 and NQO1 levels in the JB6 P+ cell line; at higher doses, apigenin reduces the expression of DNMT1 and DNMT3b	[91]
3,3-Diindolylmethane	Cruciferous vegetables	DNMT1, DNMT3aDNMT3b	3,3-Diindolylmethane reverses CpGs’ methylation for the first 5 CpGs of the NRF2 promoter, which correlates with reduced mRNA expression of DNMT1, 3a, and 3b; enhances NRF2 and NQO1 levels in prostate cells	[92]
Taxifolin	Olive oil, grapes, citrus fruits, onions	DNMT1, DNMT3aDNMT3b	Taxifolin activates the NRF2 antioxidant pathway by inhibiting DNMT1, DNMT3a, DNMT3b, HDAC1, HDAC3, and HDAC8 and reversing methylation at the first 15 CpGs of the NRF2 promoter; the expression of HO-1 and NQO1 is also increased in JB6 P+ cells	[37]

## Abbreviations

AhR—aryl hydrocarbon receptor; ALS—amyotrophic lateral sclerosis; ARE—antioxidant response element; CAT—catalase; CBP—CREB-binding protein; CNC—cap “n” collar family; COPD—chronic obstructive pulmonary disease; Cul3—Cullin3; CUR—curcumin; DIM—3,3′-Diindolylmethane; DNMTs—DNA methyltransferase; FX—fucoxanthin; GPx—glutathione peroxidase; HDACs—histone deacetylase; HFD—high-fat diet; HO-1—heme oxygenase; Keap1—Kelch-like ECH-associated protein 1; MMP—matrix metallopeptidase; NF-kB—nuclear factor kappa; NQO1—NADPH quinone oxidoreductase; NRF2—nuclear factor erythroid 2-related factor 2; PKC—protein kinase C; Res—resveratrol; ROS—reactive oxygen species; SFN—sulforaphane; sMaf—small Maf; SNP—single-nucleotide polymorphism; SOD—superoxide dismutase; TCM—traditional Chinese medicine; TET—ten-eleven translocation; TGF-β—transforming growth factor; TIIA—Tanshinone IIA; UA—ursolic acid; γ-TmT—γ-tocopherol-rich mixture of tocopherols.
